# A Novel Picosecond Pulse Generation Circuit Based on SRD and NLTL

**DOI:** 10.1371/journal.pone.0149645

**Published:** 2016-02-26

**Authors:** Jianming Zhou, Qiuyuan Lu, Fan Liu, Yinqiao Li

**Affiliations:** 1 School of Information and Electronics, Beijing Institute of Technology, Beijing, 100081, China; 2 Institute of Telecommunication Satellite, CAST, Beijing, 100094, China; National Research Council, ITALY

## Abstract

Because of the importance of ultra-wideband (UWB) radar in various applications, short pulse generation in UWB systems has attracted a lot of attention in recent years. In order to shorten the pulse, nonlinear transmission line (NLTL) is imported, which expands the application of step recovery diode (SRD) for pulse generation. Detailed analysis and equations for this SRD and NLTL-based pulse generation are provided and verified by simulation and experimental results. Factors that could cause pulse waveform distortions are also analyzed. The generator circuit presented in this paper generates 130ps and 3.3V pulse, which can be used in UWB radar systems that require sub-nanosecond pulses.

## Introduction

Nowadays, UWB radars are finding many applications in the areas of medical imaging, non-destructive testing, subsurface identifying, distance measuring, automobile traffic control and security systems [1–6], by transmitting high-intensity pulses and analyzing the reflection signals. Initially, the frequency spectrum of the UWB radar signals can be extended from low frequency to ultra-high frequency, so that it is highly penetrating and has great resolution. Furthermore, the UWB radar has low spectral density and is quite interference-proof, which is very helpful in short-range transmission. Therefore, this radar technology has attracted a lot of researchers and concerned industries across the globe to use it in any and every way for academic, business, and research purposes since the Federal Communications Commission (FCC) made the use of UWB license-free in 2002 [7].

To achieve a reliable sensing performance, however, it is necessary to develop a pulse generator with sufficient peak power and the shortest possible rise-time. Various approaches have been proposed for the design of a pulse generator for radar sensor application. CMOS integrated circuits-based pulse generators are developed to attain flexibility in controlling the pulse shape [8, 9]. However, this method has a long development time and high design cost because some of the components are customized. Other off-the-shelf designs include avalanche transistors [10], tunnel diodes [11], nonlinear transmission lines (NLTLs) [12], photoconductive switches [13], bipolar transistors [14], field effect transistors (FETs) [15], and SRDs [16–24].

Wang *et al*. [10] reported that an ultra-wideband nanosecond pulse was designed based on the avalanche effect of the avalanche transistor, where the pulse full width is 890ps and pulse amplitude is -11.2V. However, the maximum pulse repetition frequency by this method is limited, and the transistor lifetime is short. In Ruai and Konishi’s study [11], a resonant tunnel diode was utilized to produce very short pulses for switching and harmonic generation applications, but the output pulse amplitude was limited, generally speaking, not greater than 1V. Among the off-the-shelf designs, the SRD has been used as a key device because it can be easy to fabricate, cost-effective and capable of generating pulses with sharp transition edge. With the progress in electronics, the width of the narrow pulses generated via SRD is limited and cannot meet further requirements on bandwidth due to the constraints on transit time and the carrier’s lifetime. Later on, NLTL was introduced to the narrow pulse circuit.

This paper proposes a method which can generate picosecond pulses by using both SRD and NLTL. SRD is the key narrow pulse-generating component used to generate 220ps narrow bands and then NLTL is used to further narrow the width to 130ps, with the amplitude being 3.3V.

The rest of the paper is organized as follows: Section II presents the pulse generator circuit design, which consists of SRD structure, circuit generating and simulation. Section III discusses the principle of NLTL. In Section IV, we discuss our experimentation/evaluation procedure and analyze the results achieved. Section V finally concludes the paper.

## Circuit Design

### 2.1 SRD structure

**Fig 1:**
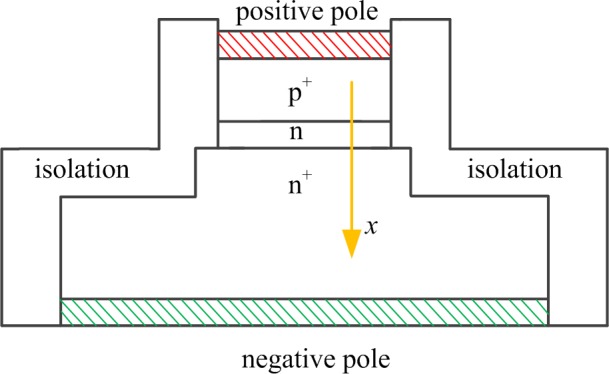
Impurity profile of general SRD.

SRD is a PN junction diode whose impurity is distributed unusually, as shown in Fig 1. Between the high doping P^+^ layer and the high doping N^+^ layer is a low doping N-type layer. It is a typical slowly varying junction structure. When SRD converts from forward exciting voltage to negative exciting voltage, a strong backward current flows continuously until it terminates at a time instant, forming a steep step voltage. The narrow pulse can be generated in this way.

The principles for this phenomenon are as follows:

1. When SRD is in the positive bias, both sides of the PN junction are infused with many minority carriers. The special impurity distribution in SRD facilitates the increase in the injected minority carriers, and creates the retarding fields at both sides of the junction which can impede the proliferation of minority carriers, thus concentrating a large number of minority carriers in the narrow regions near the junction. Furthermore, the lifetime of the minority carriers is so long that they cannot be recombinant during the period of positive bias.
2. When the positive bias converts to the negative bias, these stored minority stored will flow in the direction opposite to the injection, forming a strong backward current. When all minority carriers are extracted, the backward current will be reduced to an extremely low level suddenly, cutting off the diode and forming the step voltage.

### 2.2 Pulse-generating circuit

**Fig 2:**
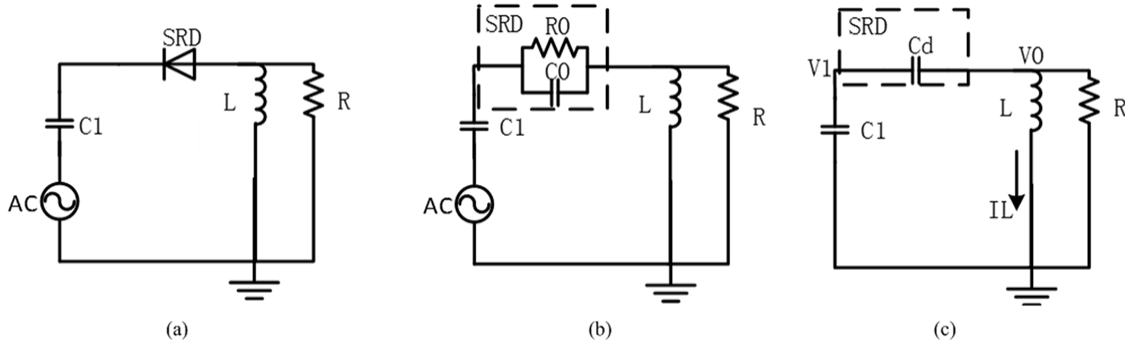
Pulse producing circuit based on SRD and equivalent circuit. (a) Principle chart (b) Equivalent circuit during conduction (c) Equivalent circuit during cut-off.

The SRD pulse-generating circuit is shown in Fig 2(A). SRD can be treated equivalently as a small resistance and a large capacitance when the forward direction is conducting and its on-state voltage is about 0.5V. The on-state equivalent circuit is shown in Fig 2(B). In the case of the SRD conversion from the forward to the backward bias, SRD is still on at a voltage of about 0.3V and the current is in the reverse direction before the SRD junction charge is depleted. Furthermore, SRD can be equivalently seen as a variable capacitance at reverse cut-off, and the equivalent circuit is shown in Fig 2(C).

The time domain response of the circuit since the instance of the SRD cut-off will be analyzed as follows. To facilitate analysis, consider that the capacitance value *C*_d_ is constant. The voltages at both sides of SRD are the on-state voltages, when SRD is on and stable. In Fig 2(C), according to the Kirchhoff theorem, we have:

$$C_{1}\frac{dV_{1}}{dt}+I_{L}+\frac{V_{0}}{R}=0$$ (1)

$$L\frac{dI_{L}}{dt}=V_{0}$$ (2)

$$C_{1}\frac{dV_{1}}{dt}=C_{d}\frac{d(V_{0}-V_{1})}{dt}$$ (3)

Eliminating *V*_1_ and *I*_L_ in the above equation yields the third-order differential equation of the voltage *V*_0_ at both sides of SRD:

$$\frac{C_{1}C_{d}}{C_{1}+C_{d}}\frac{d^{2}V_{0}}{dt^{2}}+\frac{dV_{0}}{Rdt}+\frac{V_{0}}{L}=0$$ (4)

Consider that the values of *V*_0_, *V*_1_ and *I*_L_ at the time instance of zero are equal to *V*_0(0)_, *V*_1(0)_, and *I*_L(0)_, respectively, then the boundary condition in Eq 4 is:

$$\left\{ \begin{array}{l} V_{0}\left(0\right)=V_{0}\left(0\right)\\ \frac{dV_{0}\left(0\right)}{dt}=-\frac{C_{1}+C_{d}}{C_{1}C_{d}}\left(I_{L}\left(0\right)+\frac{V_{0}\left(0\right)}{R}\right) \end{array}\right.$$ (5)

According to the infinitesimal analysis principles, *V*_0_ can be computed using Eq 4 and Eq 5 when the values of *C*_d_, *L*, *C*_1_ and *R* are known as Eq 6:

$$\begin{array}{l} V_{0}\left(t\right)=\frac{-\frac{C_{1}+C_{d}}{C_{1}C_{d}}\left(I_{L}\left(0\right)+\frac{V_{0}\left(0\right)}{R}\right)-V_{0}\left(0\right)x_{2}}{x_{1}-x_{2}}e^{{\text{x}}_{1}t}\\ +\frac{-\frac{C_{1}+C_{d}}{C_{1}C_{d}}\left(I_{L}\left(0\right)+\frac{V_{0}\left(0\right)}{R}\right)-V_{0}\left(0\right)x_{1}}{x_{2}-x_{1}}e^{{\text{x}}_{2}t} \end{array}$$ (6)

Where $x_{1}=\frac{-\frac{C_{1}+C_{d}}{C_{1}C_{d}R}+\sqrt{\frac{{\left(C_{1}+C_{d}\right)}^{2}}{{\left(C_{1}C_{d}R\right)}^{2}}-\frac{4\left(C_{1}+C_{d}\right)}{C_{1}C_{d}L}}}{2}$, $x_{2}=\frac{-\frac{C_{1}+C_{d}}{C_{1}C_{d}R}-\sqrt{\frac{{\left(C_{1}+C_{d}\right)}^{2}}{{\left(C_{1}C_{d}R\right)}^{2}}-\frac{4\left(C_{1}+C_{d}\right)}{C_{1}C_{d}L}}}{2}$

### 2.3 Circuit simulation

**Fig 3:**
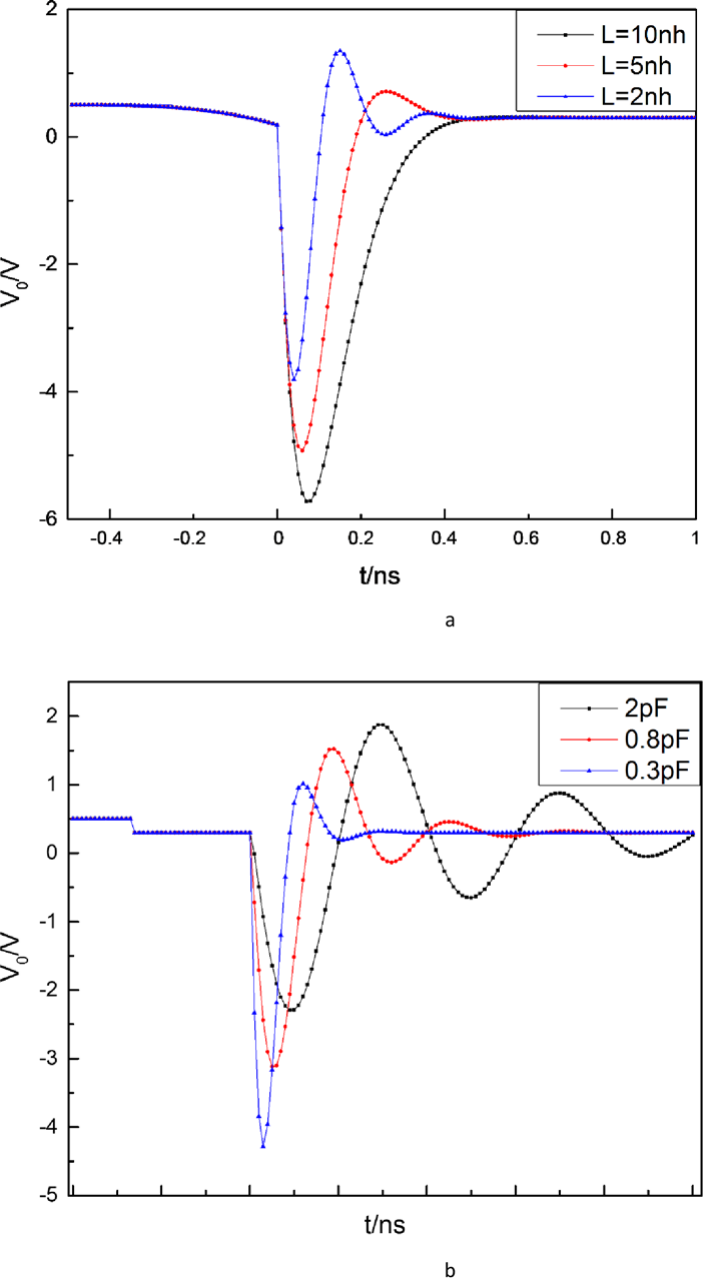
Simulation curves of pulse production circuit. (a) *C*_d_ = 0.8pF, *C*_1_ = 1μF, *R* = 50Ω (b) *L* = 0.2nH, *C*_1_ = 1μF, *R* = 50Ω.

In order to analyze the influence of *C*_d_, *L*, *C*_1_ and *R* on the *V*_0_, we design simulations in Matlab, as shown in Fig 3.

Fig 3(A) shows the amplitudes of *V*_0_ when *L* is 2nH, 5nH, and 10nH, respectively, for given values of *C*_d_ = 0.8pF, *C*_1_ = 1μF, and *R* = 50Ω. Fig 3(B) presents the amplitudes of *V*_0_ when *C*_d_ is 0.3pF, 0.8pF, and 2pF, respectively, for given values of *L* = 2nH, *C*_1_ = 1μF, and *R* = 50Ω. From Fig 3(A) and Fig 3(B), it can be concluded that the width, amplitude and shape of the generated pulse is largely dependent on the exciting inductance *L* and *C*_d_. When other parameters are given, a small value of *L* means that the pulse width is narrow, the amplitude is small, and the pulse ringing is serious. When other parameters are given, a small value of *C*_d_ means that the pulse width is narrow, the amplitude is large, and the pulse ringing is slight. This conclusion indicates that the SRD with small junction capacitance (*C*_d_) is recommended for the circuit design, and the value of *L* should be chosen taking into account pulse width and amplitude.

## NLTL Circuit

**Fig 4:**
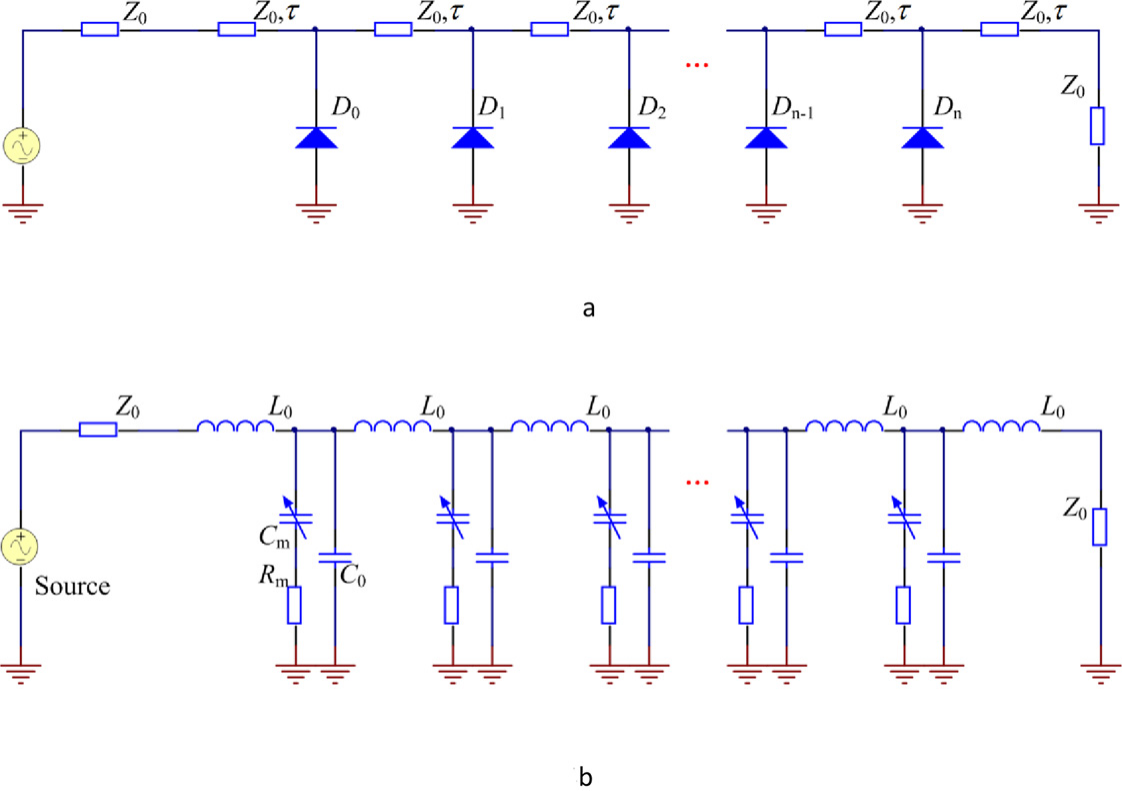
Structure and equivalent circuit of NLTL. (a) Principle diagram (b) Equivalent circuit.

Nonlinear transmission line (NLTL) is in a high impedance TEM uniform load on a transmission line consisting of varactor ladder network, which encompasses several micro-strip transmission lines with an interval of d [25]. Between the micro-strip transmission lines are the reverse bias Schottky diode, whose junction capacitance varies with the value of reverse bias. The structure of NLTL is shown in Fig 4(A). According to the micro-strip line principle, each section of the lossless micro-strip transmission line can be equivalently seen as a combination of distributed inductance and distributed capacitance. Fig 4(B) shows the equivalent circuit of NLTL, where *L*_0_ and *C*_0_ denote the distributed inductance and distributed capacitance of the lossless micro-strip transmission line, respectively, *C*_m_(V) and *R*_m_ denote the junction capacitance and the parasitic series resistance of the Schottky diode, respectively.

The network in Fig 4(B) can be seen as a low-pass filter, and the cutoff frequency is:

ωper=2L0[C0+Cm(V)]¯ (7)

The operating frequency of NLTL is upper bounded by the cutoff frequency of the Schottky diode. Consider that *R*_m_ = 0, namely, the micro-strip line is lossless, and the voltage of the next node is:

$$V_{o}\left(t\right)=V_{in}\left[t-T\left(V\right)\right]$$ (8)

Where $T\left(V\right)=\sqrt{L_{0}\left[C_{0}+C_{m}\left(V\right)\right]}$

This equation demonstrates that NLTL’s delay is related to the junction capacitance of the Schottky diode, which can be expressed as:

$$C_{m}\left(V\right)=\frac{C_{\text{j}0}}{{\left(1+\frac{V}{\phi }\right)}^{k}}$$ (9)

Where *ϕ* ≈ 0.4V (for HSMS-2820 Schottky diode), k denotes the coefficient of the diode and is usually in the range 1–3.

From Eq 1 and Eq 2, it can be known that the larger the value of reverse bias applied to the Schottky diode, the smaller the delay. For different voltages *V*_h_ and *V*_l_ (*V*_h_>*V*_l_), the delay difference of NLTL for these two voltages is:

$$\Delta T=T\left(V_{l}\right)-T\left(V_{h}\right)=\sqrt{L_{0}\left[C_{0}+C_{m}\left(V_{l}\right)\right]}-\sqrt{L_{0}\left[C_{0}+C_{m}\left(V_{h}\right)\right]}$$ (10)

Therefore, when a forward narrow pulse passes through NLTL, the pulse rise time will be shortened, and the fall time will be lengthened. To shorten the falling edge time, a grounded resistance is added to each Schottky diode in NLTL, which can steepen the falling edge.

## Measurement Results and Discussion

**Fig 5:**
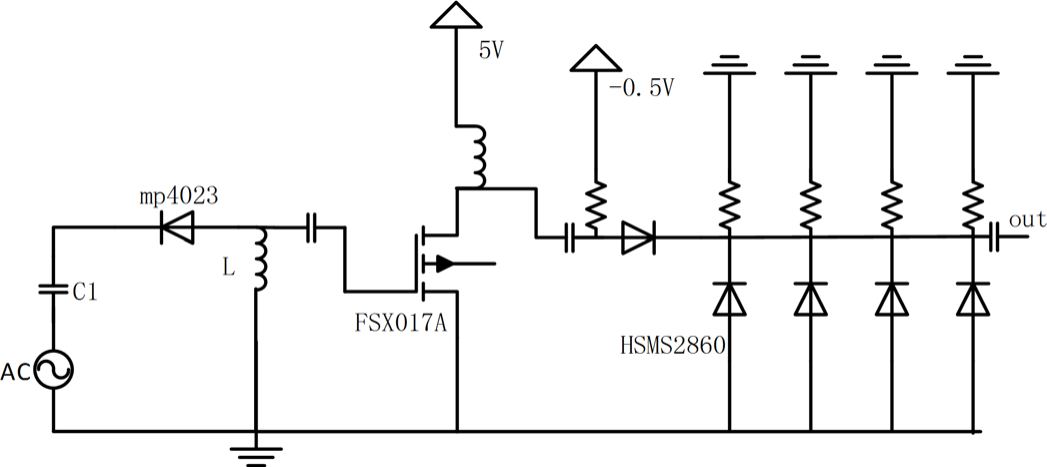
Principle diagram of pulse production circuit.

**Fig 6:**
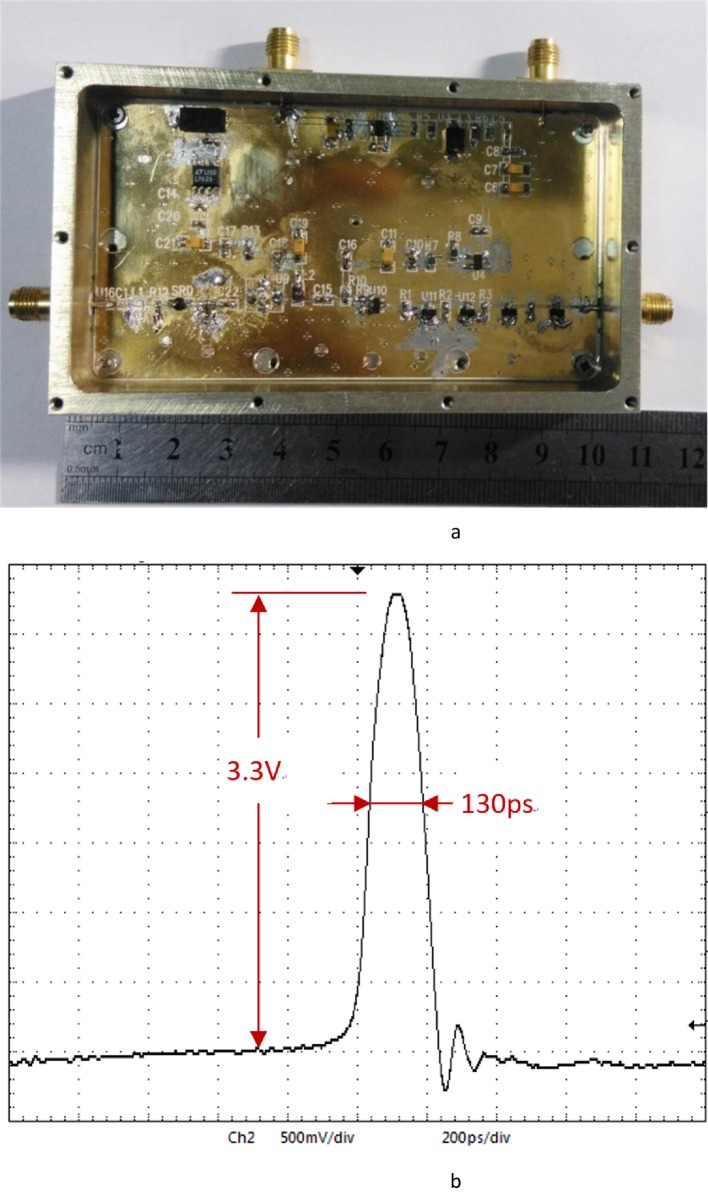
Prototype and measured waveform of designed pulse generator. (a) Prototype of designed pulse generator (b) Measured output pulse of the generator.

According to the above analysis, the narrow pulse circuit consists of the SRD pulse-generating circuit and the NLTL circuit. As shown in Fig 5, at the left is the SRD pulse generating circuit, where SRD is of the type mp4023, with junction capacitance being *C*_d_(-6V) = 0.5pF(max), transit time *t*_t_ = 50ps, carrier lifetime *t*_r_ = 12ns, *C*_1_ = 1μF, *L* = 5nH, and *R* = 50Ω. On the right is the NLTL module used to further narrow the width of the pulse generated by SRD, where *D*_1_… *D*_4_ denote the Schottky diode, *R*_1_…*R*_4_ = 50Ω, the micro-strip transmission line length is 3mm, and the characteristic impedance of the transmission line is 80Ω. According to the actual test, the waveform of *V*_out_ is shown as in Fig 6. The circuit of the pulse generator is made utilizing Teflon boards. Fig 6(A) shows the prototype of designed pulse generator, the circuit structure of which is very compact. Furthermore, the waveform is monitored by Lecroy Wave Master 8600A 6GHz DSO oscilloscope as shown in Fig 6(B). The narrow pulse amplitude is 3.3 V and the pulse width is 130ps.

## Conclusion

A narrow pulse-generating circuit is proposed based on SRD and NLTL. The influence factors in the pulse width and amplitude are discussed. Simulation results indicate that a small exciting inductance means that the amplitude of the generated pulse is small and the pulse width is narrow. The SRD with small junction capacitance will yield pulses with narrow width and high amplitude. Therefore, proper inductance and SRD with the smallest possible junction capacitance are recommended for UWB pulse-generated circuit design. To further reduce the width of the narrow pulse, the NLTL circuit is used, which can generate a narrow pulse with a half-amplitude width of 130ps and amplitude of 3.3V.
